# Supplementary material for: SNP-SNP positive interaction between MMP2 and MMP12 increases the risk of COPD
Source: PLoS One. 2024 May 21;19(5):e0301807. doi: 10.1371/journal.pone.0301807 (PMC11108124; doi:10.1371/journal.pone.0301807)
Supplement: S1 Table — (DOCX) [file pone.0301807.s001.docx]

**Table S1. The primers, restriction enzymes and length of fragments**

| **Gene** | **SNP** | **Method** | **Sequence of primers** | **Annealing temperature** | **Restriction enzyme** | **Digested fragments** | **Ref** |
| --- | --- | --- | --- | --- | --- | --- | --- |
| MMP1 | rs1799750 | RFLP | 5`- GAC TTT TAA AAC ATA GTC TAT GTT CA- 3` | 49^O^C | *Alu* I | CC allele: 269 bp  delC allele: 241, 28 bp | [29] |
|  |  |  | 5`- TCT TGG ATT GAT TTG AGA TAA GTC ATA GC - 3` |  |  |  |  |
| MMP2 | rs11646643 | RFLP | 5` - AGT GAT GAA GGT AAA GAG CAG G - 3` | 49^O^C | *Bse* NI | A allele: 178, 62 bp  G allele: 240 bp | - |
|  |  |  | 5` - GAC ACT ATA TGT ACC ACA TAC C - 3` |  |  |  |  |
| MMP2 | rs243864 | RFLP | 5’-CAC TGG TGG GTG CTT CCT TTA AC-3’ | 56^O^C | *Dde* I | T allele: 154, 33 bp  G allele: 187 bp | [30] |
|  |  |  | 5’-TGA GAT AGA AAT TGG GCA AGA CTG GTT TAC TA-3’ |  |  |  |  |
| MMP9 | rs3918253 | RFLP | 5` - GTC AGG GAA GGG AGG ACC AC– 3` | 56^O^C | *Mlu* CI | T allele: 196, 46 bp  C allele: 242 bp | - |
|  |  |  | 5` - AAC AGG TCG AGA GGA AGA GG – 3` |  |  |  |  |
| MMP9 | rs3918242 | Nested AS-PCR | 5` - GCC TGG CAC ATA GTA GGC CC– 3` | First PCR: 59^O^C  Second PCR: 62^O^C | - | First PCR amplicon: 436 bp  T allele: 296 bp  C allele: 220 bp | [28] |
|  |  |  | 5` - CTT CCT AGC CAG CCG GCA TC – 3` |  |  |  |  |
|  |  |  | 5` - AAA TTT AGC CAG GCG TGG TGG CGA AT– 3` |  |  |  |  |
|  |  |  | 5` - GCC TCC CGA GTA GCT GGT ATT ATA GTC G– 3` |  |  |  |  |
| MMP12 | rs652438 | RFLP | 5` - GGG ATA ATT TGG CTC TGG TCT TCA A– 3` | 51^O^C | *Mfe* I - HF | T allele: 181, 23 bp  C allele: 204 bp | [31] |
|  |  |  | 5` - CCA TGG GAA CCA TAG AAA AGA – 3` |  |  |  |  |
| RFLP, Restriction fragment length polymorphism; AS-PCR, Allele-specific polymerase chain reaction; bp, base pair; | | | | | | | |
