# Supplementary material for: SNP-SNP positive interaction between MMP2 and MMP12 increases the risk of COPD
Source: PLoS One. 2024 May 21;19(5):e0301807. doi: 10.1371/journal.pone.0301807 (PMC11108124; doi:10.1371/journal.pone.0301807)
Supplement: Attachment — Submitted filename: Responses to Reviewer.docx [file pone.0301807.s002.docx]

**Dear Editor,**

Here, we have provided responses to the reviewer's inquiry below.

We sincerely appreciate the reviewer's valuable feedback and the opportunity to address their inquiries. Their insights have contributed significantly to the improvement of our work.

Thank you again.

**Major:**

1. Was all p-value adjusted after multiple comparison? For example, Table 3, Table 4, and Table 5.

**Response:**

For table 3, 5 all the p-values are not for adjusted. There are only Crude ORs and its p-value calculated by logistic regression. We revised the note below the table 3 and 5, please check. Thank you.

For table 4, we calculated both crude and adjusted ORs by logistic regression. Also, p-values were calculated for both ORs. We revised the note below the table 4, please check. Thank you.

2. Page 9. Line153. Only recessive model was remaining after comparing the genotype frequency. I am confused about Table 4. The genotype frequencies of each SNP need to be calculated for both the COPD and control groups.

**Response:**

The genotype frequencies of each SNP calculated and compared for both groups with all models (recessive, dominant, multiplicative and over-dominant). However, we only included the results of selected models for each SNPs in Table 4. We used the computational and selection method for genetic model described by Horita and Kaneko. (Please see statistical analysis section.) The method calculates OR1 and OR2 based on genotype frequencies in both groups. The model was selected by intersecting the calculated OR1 and OR2 as shown in the graph below. The selected genetic model can be different for each SNPs but in this time, it was recessive for SNPs. We revised the note below the table 4, please check. Thank you.

**Minor:**

1. Table 5. After adding rs1799750, rs11646643, and rs3918253, there is no significant difference. Do all three of these SNPs collectively exhibit a risk-resistant factor for COPD?

**Response:**

After adding SNPs rs1799750, rs11646643, and rs3918253, odd`s ratio of four SNP model was increased up to 3.14, but it was not significant (p>0.05). We believe that it`s because of the small sample size. Thank you.

2. Page 11, line 169, “we found positive interaction … had increased risk for COPD” The interaction mechanism of rs243864 in MMP2 and rs652438 in MMP12 should be discussed.

**Response:**

We included some additional information related to MMP2, MMP12 and their relation in the discussion section. Please check the discussion section (line 242-249 and 256-267 of revised manuscript). Thank you.
